# Liver Regeneration: Changes in Oxidative Stress, Immune System, Cytokines, and Epigenetic Modifications Associated with Aging

**DOI:** 10.1155/2022/9018811

**Published:** 2022-07-28

**Authors:** Chaoliang Tang, Hao Chen, Lai Jiang, Lianxin Liu

**Affiliations:** ^1^Department of Anesthesiology, The First Affiliated Hospital of USTC, Division of Life Sciences and Medicine, University of Science and Technology of China, Hefei, Anhui 230001, China; ^2^Core Facility Center for Medical Sciences, The First Affiliated Hospital of USTC (Anhui Provincial Hospital), Hefei, Anhui 230001, China; ^3^Department of General Surgery, The First Affiliated Hospital of USTC, Division of Life Sciences and Medicine, University of Science and Technology of China, Hefei, Anhui 230001, China; ^4^Department of Obstetrics and Gynecology, The First Affiliated Hospital of USTC, Division of Life Sciences and Medicine, University of Science and Technology of China, Hefei, Anhui 230001, China; ^5^Department of Hepatobiliary Surgery, The First Affiliated Hospital of USTC, Division of Life Sciences and Medicine, University of Science and Technology of China, Hefei, Anhui 230036, China; ^6^Anhui Provincial Clinical Research Center for Hepatobiliary Diseases, Hefei, Anhui 230036, China; ^7^Anhui Provincial Key Laboratory of Hepatopancreatobiliary Surgery, Hefei, Anhui 230036, China

## Abstract

The regenerative capacity of the liver decreases with increase in age. In recent years, studies in mice have found that the regenerative capacity of the liver is associated with changes in the immune system of the liver, cytokines in the body, aging-related epigenetic modifications in the cell, and intracellular signaling pathways. In the immune system of the aging liver, monocytes and macrophages play an important role in tissue repair. During tissue repair, monocytes and macrophages undergo a series of functional and phenotypic changes to initiate and maintain tissue repair. Studies have discovered that knocking out macrophages in the liver during the repair phase results in significant impairment of liver regeneration. Furthermore, as the body ages, the secretion and function of cytokines undergo a series of changes. For example, the levels of interleukin-6, transforming growth factor-alpha, hepatocyte growth factor, and vascular endothelial growth factor undergo changes that alter hepatocyte regulation, thereby affecting its proliferation. In addition, body aging is accompanied by cellular aging, which leads to changes in gene expression and epigenetic modifications. Additionally, this in turn causes alterations in cell function, morphology, and division and affects the regenerative capacity of the liver. As the body ages, the activity of associated functional proteins, such as CCAAT-enhancer-binding proteins, p53, and switch/sucrose nonfermentable complex, changes in the liver, leading to alterations in several signaling pathways, such as the Hippo, PI3K-Akt, mTOR, and STAT3 pathways. Therefore, in recent years, research on aging and liver regeneration has primarily focused on the immune system, signaling pathways, epigenetic changes of senescent cells, and cytokine secretion in the liver. Hence, this review details the roles of these influencing factors in liver regeneration and impact of aging-related factors.

## 1. Introduction

Liver cells are capable of rapid regeneration and repair. When partial liver resection or liver cell necrosis occurs, the remaining liver cells are regenerated and repaired, restoring not only normal liver function but also normal liver weight and volume. The regeneration of the liver is strictly regulated, with hepatocyte proliferation ceasing once the liver/body weight ratio returns to normal [1, 2]. Adult hepatocytes can reenter the cell cycle quickly after partial liver resection, but they proliferate at a low rate for homeostasis. The replacement rate of hepatocytes is relatively low during homeostasis, with only approximately 20% replaced within a year [3]. The 2/3 hepatectomy in a mouse model developed by Higgins and Rogers in 1931 is considered the best model for liver regeneration. When 2/3 of the mouse liver is resected, the remaining hepatocytes transition from the G0 to G1 phase and rapidly reenter the cell cycle, restoring the liver to its normal weight [4]. When this ratio is reached, the associated signaling molecules and pathway activity change to regulate normal liver function. The loss of this regulatory ability leads to abnormal proliferation and cancer development [5]. However, the molecular mechanism of this cell cycle transition is still unknown.

Recently, liver regeneration divided into three basic processes: (a) preparation for liver regeneration that involves the secretion of cytokines and growth factors, which activate the associated signaling pathways and change cell activity; (b) liver recovery, which initiates after signaling pathway activation, the cell cycle of quiescent hepatocytes changes, and hepatocytes initiate proliferation to repopulate the damaged liver; and (c) termination of liver regeneration, which includes the termination of hepatocyte proliferation by regulating signaling pathways after the liver/body weight ratio returns to the original level [6].

As the body ages, changes in several mechanisms of the body occur in varying degrees, which affect the liver's regeneration capacity. Most recent studies are based on studying such changes to observe their effects on the mechanisms of liver regeneration. The body's immune system, metabolic system, cell contents, cell microenvironment, and gene expression change as the body ages. However, to date, there is still no clear evidence that fully explains the decline of liver regeneration capacity with aging.

## 2. The Oxidative Stress and Regeneration of the Aging Liver

ROS are generated in response to tissue injury, and their induction is required for tissue regeneration. Oxidative stress is a complex defense mechanism that involves multiple signaling pathways and related proteins. The intracellular redox state is closely related to the signal transduction pathways involved in cell apoptosis, proliferation, and differentiation [7]. Nuclear factor E2-related factor 2 (NF-E2-related factor 2, Nrf2) is an intracellular transcription factor that is highly sensitive to oxidative stress, mainly in metabolic organs, such as the liver [8]. It was discovered that after partial hepatectomy (PH) in mice, large amounts of ROS were produced in the liver, causing oxidative stress. Compared with the control group, after PH, the liver regeneration of Nrf2 knockout mice was significantly delayed, the rate of hepatocyte apoptosis was doubled, and mitogen-activated protein kinase signaling was transduced in the regenerated liver. The signal transduction of phosphatidylinositol 3-kinase/protein kinase B (PI3K)/protein kinase B (PI3K/Akt) was attenuated, and the number of regenerated hepatocytes was significantly reduced at 60 h after PH [9, 10]. The expression of genes that are closely related to function was downregulated. These results suggest that Nrf2 plays an important role in the regeneration of liver tissue repair.

**Figure 1:**
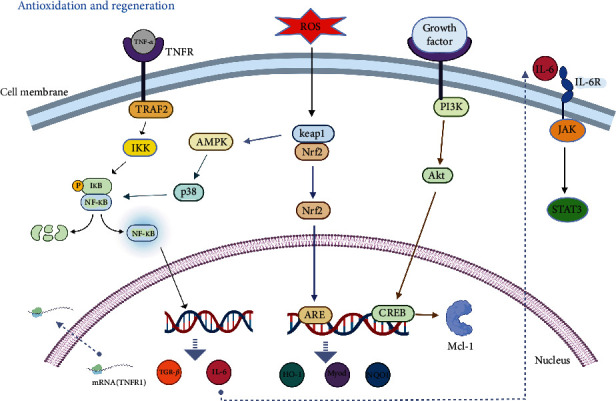
The diagram of ROS and its related pathway in liver regeneration.

Nuclear factor *κ*B (NF-*κ*B) is an important transcription factor that helps the body maintain redox balance [11]. Under normal conditions, inactive NF-*κ*B is mainly composed of p50 and p65 subunits, as well as the inhibitor of NF-*κ*B (I*κ*B), which is bound in the cytoplasm. NF-*κ*B can be activated by a variety of stimuli, and the I*κ*B kinases (I*κ*B kinase a and I*κ*B kinase b) promote the phosphorylation and degradation of I*κ*B, resulting in the separation of I*κ*B from NF-*κ*B. The activated NF-*κ*B is then transferred from the cytoplasm to the nucleus, where it participates in cell proliferation and survival, allowing the body to function normally. NF-*κ*B regulates liver regeneration by upregulating the expression of interleukin 6 (IL-6) and hepatocyte growth factors (HGF) [12, 13]. NF-*κ*B activation is one of the earliest responses detected at the onset of liver regeneration after liver injury or PH, suggesting that NF-*κ*B is a key factor capable of preventing apoptosis and regulating hepatic cell cycle progression during liver regeneration (Figure 1).

The PI3K/Akt pathway is associated with physiological processes, such as cell antiapoptosis, antioxidation, and protein synthesis. The PI3K/Akt pathway plays an important role in the regenerative activity of the liver, and Akt inactivation can lead to the inhibition of hepatocyte proliferation after PH [14]. Jackson et al. found that PI3K was activated as soon as PH was performed [15]. The use of PI3K inhibitors or selective inhibition of PI3K subunits using siRNA technology was found to significantly reduce liver regeneration. The inhibition of PI3K resulted not only in a reduction of Kupffer cells and macrophages in the regenerating liver but also in secretory dysfunction of Kupffer cells and macrophages, resulting in decreased cytokine production and a decreased hepatocyte proliferation rate. This result suggests that activation of the PI3K/Akt pathway plays a critical role in the early stages of liver regeneration after PH.

## 3. The Immune System and Regeneration of the Aging Liver

**Figure 2:**
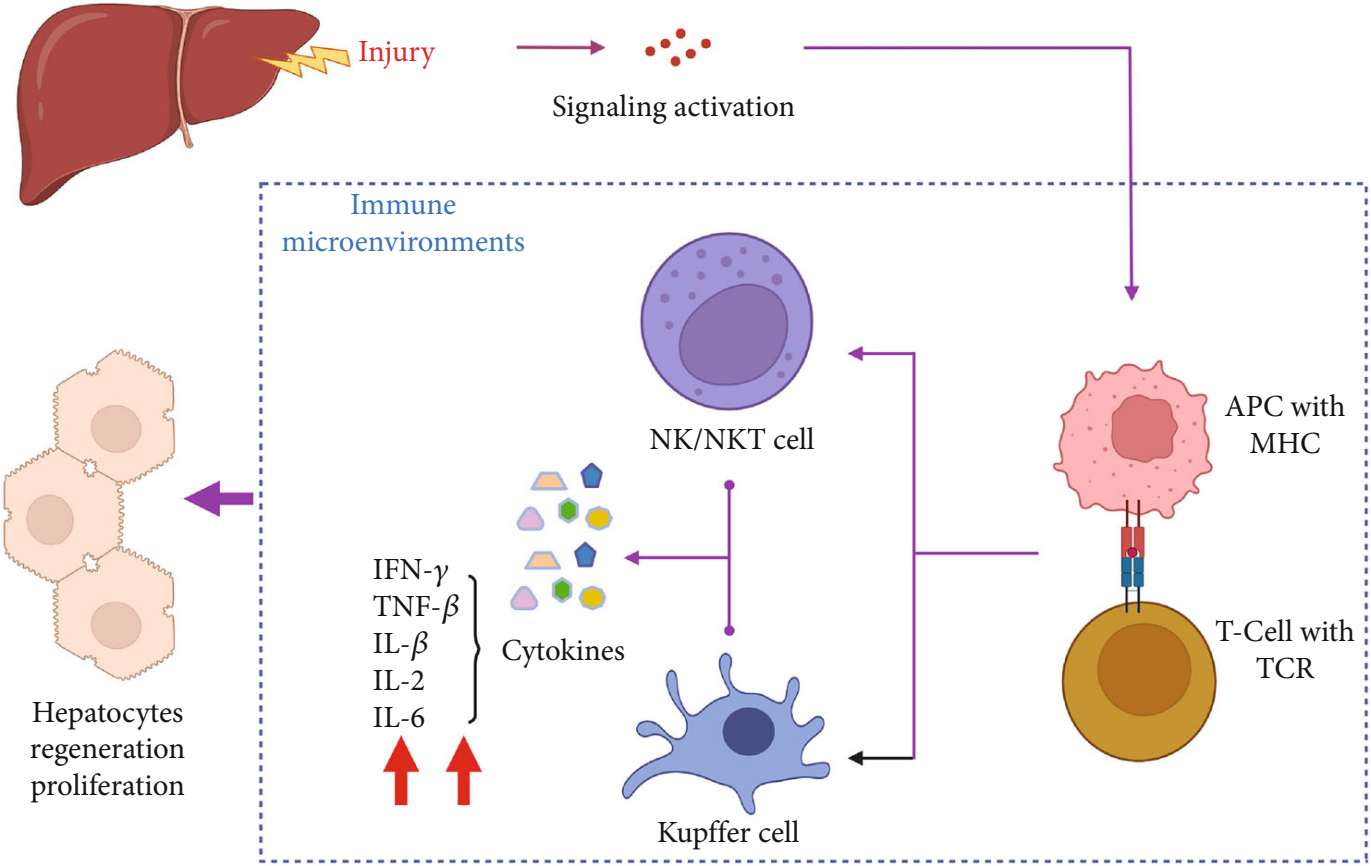
Regulation of the immune microenvironment by TCR*β* rearrangement and subsequent effects on hepatocyte regeneration.

In recent years, increasing evidences have shown that the liver is a metabolic organ and an “immune system organ” [16]. Most of the cells in the liver are hepatocytes, accounting for about 78%–80% of the total liver tissue, whereas the nonparenchymal cells only account for 5%–6% of the total liver tissue [17–21]; and the remaining 14%–17% of the liver tissue comprise the cellular components in the extracellular space [17]. The nonparenchymal cells are of various types, such as Kupffer cells, lymphocytes, cholangiocytes, endothelial cells, and stellate cells [22]. Endig et al. reported that lymphocytes have significant effects on liver injury and cancer but can also protect mice from acute injury caused by chronic liver failure and support proliferation of hepatic progenitor cells during chronic liver injury. These findings highlight the body's immune system to be tightly and specifically regulated to balance its immune surveillance and reduce the risk of cancer. More importantly, the inhibition of lymphotoxin-*β* receptor signaling may be an interesting approach to prevent tumor development in patients with chronic liver disease with high levels of lymphotoxin-beta [23]. Various lymphocytes in the liver play different roles in liver regeneration. In 2006, Castellaneta et al. found that interstitial Langerhans-type dendritic cells are essential in regulating local immunity during liver regeneration, proposing that this phenomenon may be associated with the regulation of estrogen-mediated immunity and hepatocyte proliferation. Recent studies have demonstrated the existence of a large number of natural killer (NK) cells in the liver and found that liver-resident NK (LrNK) cells inhibit T cell function in the liver during viral infection through the programmed cell death protein 1–programmed death-ligand 1 interaction. The results also revealed that LrNK cells play a significant regulatory role in liver injury [24]. As aging progresses, the immune system of the liver further changes, exhibiting distinct inflammation. Simultaneously, immune cell infiltration is increased [25], which increases the number of lymphocytes, resulting in an increased expression of the proinflammatory cytokines, including interferon-gamma (IFN-*γ*), interleukin-beta (IL-*β*), IL-6, tumor necrosis factor-alpha (TNF-*α*), and IL-2, and the anti-inflammatory cytokines, which include transforming growth factor-beta (TGF-*β*) and IL-10 [26–29] (Figure 2). Singh et al. found that the elevated levels of IFN-*γ* in the aging liver lead to phosphorylation of STAT1 (an H3 deacetylase that upregulates the postoperative levels of glucose (Glu) and triglycerides (TG), which play an essential role in liver regeneration), which activates p21 protein that inhibits cyclin-dependent kinases (CDKs), thereby inhibiting cell cycle progression [30]. In addition, it was found that liver inflammation became more severe as aging progressed. These results indicate that cytokines play an essential role in regulating liver aging via regulatory proteins. In recent years, it has been reported that IL-6 and TGF-*β* have positive regulatory effects on the *in vitro* growth and regeneration of hepatocytes [31–33], although attributed to the excessive inflammatory response *in vivo*, resulting in a decreased liver regeneration capacity [34].

## 4. Metabolism and Regeneration in the Aging Liver

**Figure 3:**
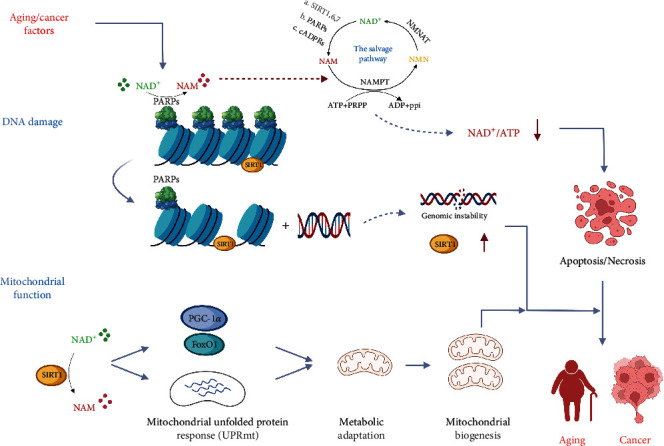
The decrease of NAD^+^ not only affect the expression of mitochondria-related genes in the nucleus but also can protect mitochondria by regulating the activities of oxidase and reductase in the cytoplasm. Reduced levels of NAD^+^ also increase the stability of the genome through affecting the activity of PARPs and Sirtuins.

Liver regeneration largely depends on the energy required to regenerate the hepatic lobules [6]. However, aging is often accompanied by changes in metabolism. Cholesterol accumulation, glycogen storage disorders, and disruption of ketone body synthesis, accompanied by the downregulation of adenosine triphosphate (ATP) and upregulation of adenosine diphosphate and reactive oxygen species, occur in the aging liver [35]. Kachaylo et al. demonstrated transient regeneration-associated steatosis (TRAS) during liver regeneration and found this to be indispensable for liver repair [3, 36]. Lipid synthesis is a critical process in the early stages of liver regeneration. Kachaylo et al. demonstrated that the downregulation of phosphatase and tensin homolog promotes the degradation of TRAS-derived lipids, thereby enhancing the regenerative capacity of the liver [37]. If the production of these lipids is disturbed, the regenerative ability of the liver becomes significantly impaired [3, 36]. Mastrodonato et al. and Fernandez-Rojo et al. showed that the specific knockout of caveolin-1 protein expression (which affects lipid accumulation and is important for maintenance of glucose homeostasis) results in a significantly impaired liver regeneration and repair in mice [38, 39]. Furthermore, SIRT1 is critical for controlling cellular metabolism since it facilitates catalytic processes that restore cellular energy homeostasis in conditions with low energy availability (low ATP) [40, 41]. However, SIRT1 overexpression has adverse effects on liver regeneration, manifesting as poor survival and impaired proliferation after hepatectomy. Interestingly, a recent study by Jin et al. showed that decreased expression of SIRT1 in aging mice was associated with impaired liver regeneration (Figure 3). Additional SIRT1 protein injections to normalize their *in vivo* levels significantly upregulated the regenerative capacity of aging mice, indicating that restoring normal metabolic processes in aging mice could improve their regenerative capacity [42]. Although liver metabolism still changes with aging, restoring this disordered metabolic phenomenon to normal can significantly improve the poor regeneration of the aging liver [35, 42].

## 5. Gene Expression and Regeneration in the Aging Liver

**Figure 4:**
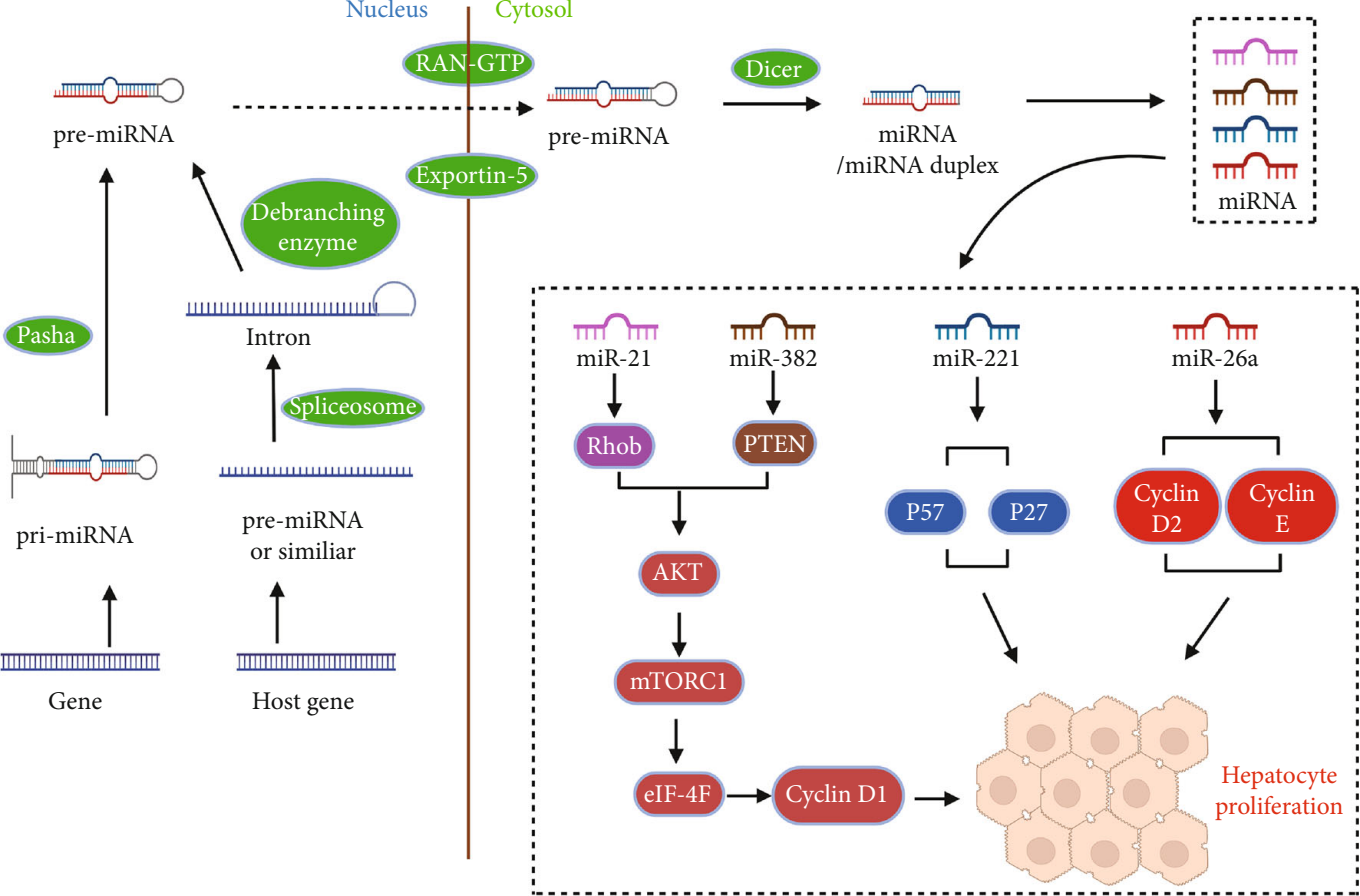
The molecular mechanisms of certain miRNAs to regulate liver regeneration.

Along with the growth and development of the liver, gene expression in hepatocytes is constantly changing [43]. Elias et al. analyzed the RNA transcriptomes of livers from 5-, 24-, and 36-month-old mice and found 56 miRNAs whose expression profiles changed with age. These included a cluster of 18 miRNAs that were upregulated 50 and 1,000 folds at 24 and 36 months of age, respectively [44]. In addition, senescent cells express p16Ink4a, a CDK inhibitor and tumor suppressor. This pathway leads to impaired cellular regeneration in senescent organisms and may be a hallmark of senescence [45]. Aging is associated with replicative senescence, and p16 levels increase with the aging of most mammalian tissues. However, p16 expression is increased after hepatectomy in mouse models, and the expression of downstream molecular target cyclin D1 decreased, suggesting that the mechanism of hepatocyte cell cycle declines gradually with age, followed by cell cycle arrest [46]. Both hepatocyte growth factor and receptor tyrosine-protein kinase MET (cMET) are essential for liver regeneration [47]. In a mouse model of partial liver resection, HGF and cMET expression were downregulated in aging mice [48], suggesting that a decreased expression of HGF and cMET is one of the mechanisms of impaired liver regeneration in aging mice. Cell aging is often accompanied by changes in the chromosomal structure, increased complexity of cellular contents, DNA damage, inhibition of CDK activity, and increased autophagy [49]. Some genes originally located in the heterochromatin regions expressed and regulated the progression of cell cycle after contact with the inhibited state during aging. For example, the members of the miR-465 family are present in a low expression state in young mice but begin transitioning to a high expression state with age. Elias et al. transfected the hepatocyte cell line AML12 with the miR-465 family members, showing a 40% reduction in the mRNA levels of growth hormone receptors and a 25% reduction in Kit1 and PPP2R3C [44]. The result confirms that the miR-465 family members are continuously upregulated with aging and attenuate the expression of related genes in the glucocorticoid signaling pathway, thereby affecting the corresponding signaling pathways and further affecting the liver regeneration capacity. Recent studies showed that miRNAs are closely associated with liver regeneration. Most miRNAs play a regulatory role, and Kren et al. revealed the role of miRNAs in the regulation of c-Myc and p53, which leads to changes in gene expression during liver regeneration [50]; an increasing number of miRNAs have been found that regulate liver regeneration, including miR-21 and miR-34 [51, 52]. Raschzok et al. studied the expression of 323 miRNAs after hepatectomy and found that the expression levels of 29 miRNAs were significantly changed [53]. Among these, seven miRNAs (miR-33, miR-153, miR-298, miR-301b, miR-489, miR-743b, and miR-883) were upregulated but did not attain a peak until 24 h after resection, which is consistent with DNA replication. The result showed that these seven miRNAs play an essential role primarily during the G1/S phase in the early stage of liver regeneration (Table 1). Further studies by Raschzok et al. found that the target genes of these miRNAs are CDK6, RAP2A, TNF, CCND1, and MAP3K1. Some genes are also involved in regulating the signaling pathways that affect liver regeneration. For example, Castro et al. found that miR-19a, miR-21, and miR-214 regulate the phosphatase and tensin homolog (a negative regulator of the phosphatidylinositol 3-kinase (PI3K)–protein kinase B (Akt) survival pathway) [54] (Figure 4). Due to changes in chromatin structure caused by cellular aging, the expression of intracellular genes becomes more complex. Hence, several studies have found that miRNAs expressed during aging play major roles in regulating the regenerative capacity of the aging liver. Pibiri et al. studied the RNA levels of mice at different time intervals after liver resection. They reported that the gene expression levels of aging mice were significantly different from those of young mice. Furthermore, the time of expression of some growth factors and cytokines was delayed in aging mice than in young mice [55]. In addition, recent studies revealed that the changes in epigenetic modifications in senescent cells could regulate hepatocyte proliferation as aging progressed, which may explain the decreased regenerative capacity of the aging liver. Sen et al. found through high-throughput screening that the histone acetyltransferase p300 is a key protein that regulates aging, induces the formation of new super-enhancers, and promotes the expression of aging-related genes [56]. By knocking out the p300 gene, they found that cell aging was delayed and replicative senescence was alleviated, suggesting the use of p300 as a potential target for treating aging-related lesions. DNA methylation plays an extremely important role in gene expression and silencing, including the degree of DNA methylation, which changes with age. Age-induced changes in methylation can lead to several aging-related diseases [57]. Kaji et al. found that knocking out the expression of DNA methyltransferase results in severe DNA damage, cell cycle arrest, senescence, and cell death. They also found that such defective or downregulated DNA methyltransferase expression impaired liver regeneration capacity [58]. Varela-Rey et al. showed that glycine-N-methyltransferase (GNMT) knockout impaired multiple signaling pathways involved in liver regeneration in mice, suggesting GNMT participation in liver regeneration and the survival and normal proliferation of hepatocytes [59]. Although there is currently abundant evidence that epigenetic changes can directly lead to impaired liver regeneration, there is still no clear evidence that epigenetic changes due to aging are the principal cause of liver damage. In summary, the changes in gene expression caused by aging are directly involved in regulating liver function, regeneration, and energy balance. These changes in gene expressions are mainly reflected as changes at the transcriptional level. The primary proteins and stably expressed genes are regulated by miRNA, thereby changing numerous liver functions. There remain several influencing factors and processes at the gene level that are yet to be discovered.

## 6. The Effect of Signaling Pathways on the Regeneration of the Aging Liver

**Figure 5:**
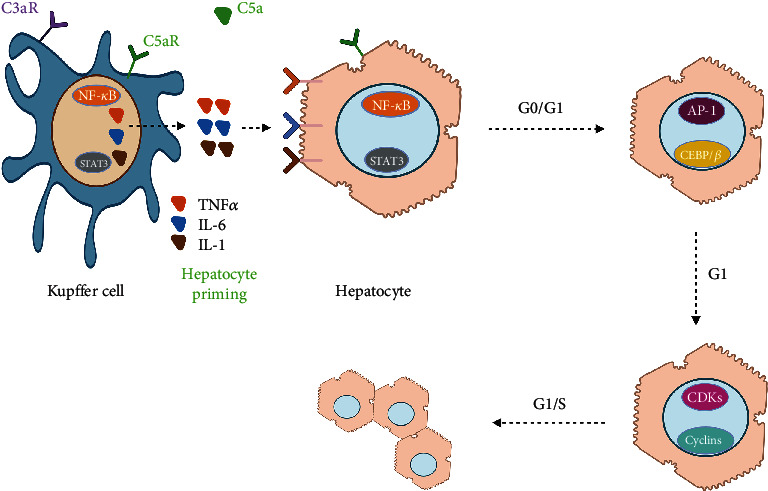
Cytokines activate NF-*κ*B and STAT-3 via corresponding receptors in hepatocytes and then initiate the transcription of immediate early genes. The final transition into G1 phase and the transcription of cell cycle genes is supported by the activation of several transcription factors.

Cell aging increases the complexity of the cellular microenvironment. Senescent cells produce cytokines, chemokines, and protein enzymes, possessing both positive and negative effects in several physiological processes, such as tissue repair, tumor development, and other processes [60–62], causing changes in cellular signaling pathways that play an indispensable role in cell development, differentiation, and proliferation [63]. During the progression of cell aging, changes occur in the interaction between some primary proteins, which alter the original function of the associated signaling pathways [64]. In the liver, TNF-*α*, IL-6, NF-*κ*B, and STAT3 were first detected in young mice after partial liver resection [65–67]. This further activated tyrosine kinase receptors, cMET, and EGF ligands, thereby activating the expression of transcription factors involved in liver regeneration [68]. The transcription factors activated after resection primarily include c-jun, C/EBPb, and cAMP response element modulator [69–72]. The liver subsequently initiates the expression of certain proteins required for the S-phase transition, including DNA polymerase A, c-myc, cdc2, and FoxM1B [73–79]. In addition to gene activation, the liver must abrogate the inhibition of the C/EBPa and E2F–Rb complexes, which inhibit E2F-dependent promoters. In the young liver, the elimination of these inhibitory effects is mediated by the PI3K-Akt signaling pathway [80]. Stocker and Heine found that 99% of hepatocytes in young mouse livers proliferated after PH, whereas only 30% proliferated in aging mouse livers after PH [81]. Fry et al. found that DNA polymerase A activity, a key enzyme in DNA replication, was significantly inhibited in the liver of aging mice after PH [73]. The expression of several cell cycle proteins (e.g., c-myc, c-fos, cdc2, and FoxM1B) is also inhibited in the liver of aging mice after PH [69, 70, 75, 77, 79]. Interestingly, most of the inhibited genes in the aging liver are regulated by the E2F family of transcription factors and inhibited by the E2F–Rb family complex [69, 82, 83]. In addition, the frequency of polyploid hepatocytes is increased in the livers of aging mice (from 7% to 10% in young mice to 30% in aging mice) [84]. Wilkinson et al. demonstrated that diploid hepatocytes proliferate faster than polyploid hepatocytes, with polyploidy inhibiting the proliferation of most hepatocytes [85]. These studies suggested that the molecular basis of the reduced proliferative response in the aging liver may be associated with the alterations of signaling pathways at the translational and/or posttranslational modification levels. Notably, the first evidence of epigenetic effects on liver proliferation in aged mice was obtained by studying the expression of C/EBPa in the livers of aging mice. The high levels of C/EBPa expression in the liver prevent the proliferation of the young liver by CDK2 inhibition [86, 87]. The detection of C/EBPa complexes in the liver of aging mice showed that aging could convert C/EBPa from CDK2 complexes to macromolecular complexes containing Rb, E2F4, and the chromatin-remodeling protein BRM (i.e., the C/EBPa–Brm complex) [75, 88]. The C/EBPa–Brm complex occupies E2F-dependent promoters, such as b-myb, cdc2, dihydrofolate reductase, and c-myc, and represses the expression of these genes after resection [75] (Figure 5). Studies have further revealed that the C/EBPa–Brm complex inhibits the expression of FoxM1B, which is necessary for normal liver regeneration [82, 83]. Therefore, changes in the signaling pathways in aging hepatocytes can lead to changes in the expression of several genes, consequently leading to decreased cellular regeneration capacity. In recent years, increased changes in signaling pathways have been discovered in the aging liver. For example, Loforese et al. found that the Hippo signaling pathway is damaged in aging mice and that inhibiting the expression of the key enzyme MST1/2 enhanced the regenerative capacity of aging hepatocytes in mice [89]. These studies showed that with the progression of aging, liver regeneration capacity decreases due to the changes in the associated cellular signaling pathways. If these changes caused by aging are reversed, the regeneration capacity of the aging liver may also be restored. It is therefore important to identify the signaling pathways by which cells proliferate and maintain their differentiated functions and to determine the impact of aging on these pathways.

## 7. Conclusion

**Figure 6:**
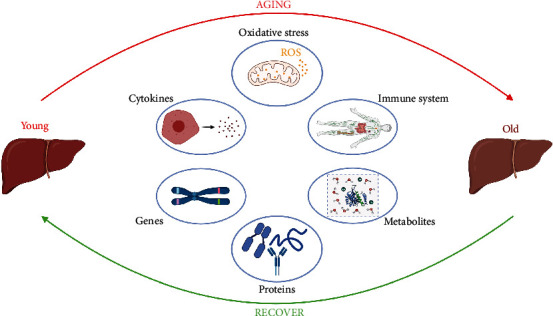
Schematic diagram of the aging-related changes in the liver. During the aging process of the liver, a series of alterations was observed in genes, proteins, metabolites, etc. The oxidative homeostasis was disrupted, thus leading to oxidative stress.

The decreased liver regeneration capacity due to aging results from many factors, such as the intracellular and extracellular changes caused by cell aging. Although no clear evidence exists to explain the mechanism of this occurrence, the notion that hepatocytes lose their ability to increase with age is challenged by some experimental evidence based on the successful *in vitro* expansion of hepatocytes [90]. Recent studies have proposed that decreased liver regeneration capacity in aging organisms is not due to replicative senescence of hepatocytes in the liver. The addition of cytokines to the culture medium *in vitro* allows isolated primary hepatocytes to enter the cell cycle, maintaining growth *in vitro* for a long time [91]. Therefore, the reduced regenerative capacity of the aging liver can likely be summarized as follows: (1) the decreased expression of cell adhesion proteins leads to weakened microstructural adaptation and p21-dependent cell cycle arrest after tissue injury [92]. (2) Changes in the secretion of cytokines, such as TGF, IL-6, and IL-1*β*, in the aging liver lead to an imbalance of the liver's internal environment, resulting in the increased complexity of cellular components in the liver and its continuous effects on hepatocyte function and proliferation. (3) Changes in the metabolic capacity of the aging liver lead to the accumulation of associated substances that affect the microenvironment of hepatocytes and compromise their proliferation. (4) Ineffective clearance of aging hepatocytes results in continuous changes in the immune system of the liver and differential secretion of cytokines and chemokine. Therefore, this regulates the accumulation of lymphocytes, such as macrophages, in the liver, further increasing the internal environment complexity of the aging liver to regulate the native cells in the liver. (5) With age, the expression of genes constantly changes and the genes that were primarily not expressed in the heterochromatin are gradually be upregulated, leading to numerous phenotypic and morphological changes in hepatocytes, which in turn leads to hepatocyte damage and decreased liver regeneration capacity (Figure 6).

Currently, the mechanisms of decreased liver regeneration capacity caused by aging have not been fully examined, but with the increase in the aging population, such studies have become increasingly urgent.

## Figures and Tables

**Table 1:** Significantly regulated miRNAs during HSC activation. References upregulated.

References	Up-regulated	Down-regulated
Guo et al. 2009b [93]	miR−**29c**, −138, −140, −**143**, −**193**, −207, −325−5p, −328, −349, −501, −872, −874	miR−15, −**16**, −20*b* −3p, −92b, −122, −**126**, −**146a**, −341, −375

Ji et al. 2009 [94]	miR-27a, −27b, −30a, −30c, −30d, −130a, −130b, −450, −455	miR-9, −19b, −301, −520b, −520c, −721

Maubach et al. 2011 [95]	Let-7b, −7c, −7e, miR-125b, −**21**, −22, −31, −132, −143, −145, −**152**, −199a, −210, −**214**, −**221**, −**222**	Let-7f, miR−10a, −**16**, −26b, −**29a**, −30*a* −5p, −30b, −30c, −30d, −99a, −122a, −125a, −**126**, −1**46a**, −150, −151, −181a, −**192**, −**194**, −**195**, −207, −296, −335, −422b, −483

Chen et al. 2011 [96]	miR-31, −34b, −34c, −125b−5p, −**143**, −145, −**152**, −**193**, −199a −5p, −199a−3p, −214, −218, −**221**, −**222**, −301a,−345−5p, −425	miR-10a−5p, −101a, −**126**, −139−5p, −**150**, −**192**, −**195**, −335, −338, −378, −450a, −497, −877

Lakner et al. 2012 [97]	miR−34c, −184, −221	miR−**16**, −19a, −**19b**, −**29a**, −**29c**, −92a, −**150**, −**194**

Raschzok et al. 2011 [53]	miR33, -153, -298, -301b, -489, -743b, -883	

Castro et al. 2010 [54]	miR19A, -**21**, -**214**	

Summary of published data regarding microRNA microarray profiling of activating primary rat HSCs. miRNAs which display an overlap in different published data sets are displayed in bold.

## Data Availability

No data were used to support this study.
